# Noninvasive Diagnosis of Irritable Bowel Syndrome via Bowel Sound Features: Proof of Concept

**DOI:** 10.14309/ctg.0000000000000017

**Published:** 2019-03-22

**Authors:** Xuhao Du, Gary Allwood, K. Mary Webberley, Andrisha-Jade Inderjeeth, Adam Osseiran, Barry James Marshall

**Affiliations:** 1 Marshall Centre, The University of Western Australia, Perth, Western Australia, Australia;; 2 North Metropolitan Health Service, Sir Charles Gairdner Hospital, Nedlands, Western Australia, Australia;; 3 School of Engineering, Edith Cowan University, Western Australia, Australia.

## Abstract

Irritable bowel syndrome (IBS) is a common and debilitating disorder estimated to affect approximately 11% of the world's population. Typically, IBS is a diagnosis of exclusion after patients undergo a costly and invasive colonoscopy to exclude organic disease. Clinician's and researchers have identified a need for a new cost-effective, accurate, and noninvasive diagnostic test for IBS.

**METHODS:**

Using a diagnostic case-control study, we explored the use of bowel sounds to characterize IBS with a view to diagnostic use. We recruited participants with an existing clinical diagnosis of IBS or healthy (asymptomatic) digestive systems. We recorded bowel sounds for 2 hours after fasting and then for 40 minutes after a standard meal.

**RESULTS:**

We here report our results including our accuracy in characterizing IBS-related bowel sounds and differentiation between participants with IBS and healthy participants. Leave-one-out cross-validation of our model developed using the first 31 IBS and 37 healthy participants gave 90% sensitivity and 92% specificity for IBS diagnosis. Independent testing using the next 15 IBS and 15 healthy participants demonstrated 87% sensitivity and 87% specificity for IBS diagnosis.

**CONCLUSIONS:**

These preliminary results provide proof of concept for the use of bowel sound analysis to identify IBS. A prospective study is needed to confirm these findings.

**TRANSLATIONAL IMPACT:**

Our belt and model offer hope of a new approach for IBS diagnosis in primary practice. Combined with screening tests for organic disease, it would offer greater confidence to patients and could reduce the burden of unnecessary colonoscopies for health care systems and patients.

## INTRODUCTION

Irritable bowel syndrome (IBS) is a debilitating functional gastrointestinal (GI) disorder with symptoms including altered bowel habits, abdominal pain, and bloating. IBS is extremely common, affecting approximately 11% of the world's population (1), and is responsible for 50% of gastroenterology clinic visits in the United States (2). In addition to direct medical costs, IBS also leads to indirect costs through lost productivity and can severely impact on an individual's quality of life (3,4).

The current gold standard for IBS diagnosis is through the Rome IV symptom-based diagnostic criteria (5). While offering positive diagnosis, these criteria are unwieldy (6) and do not have high reliability (7). In addition, a number of organic diseases share symptoms with IBS, including Crohn's disease, ulcerative colitis, and celiac disease (5). Because of similarities in clinical presentation, many physicians proceed with invasive testing to rule out organic disease before confirming a diagnosis of IBS (3,8). Initial screening would usually include baseline blood tests (including C-reactive protein) and stool tests (including fecal calprotectin and culture) for exclusion of infections, celiac disease, and inflammatory bowel disease (IBD) (5). Typically, primary care physicians also refer patients for colonoscopy and biopsy (3), although colonoscopy has been found to reveal inflammatory bowel disease in only a small percentage of patients with IBS symptoms (9).

These invasive tests are a burden to health systems, contributing to lengthy waiting lists for gastroenterological review and adding to the financial costs associated with IBS. Colonoscopies are not only unpleasant for patients but carry significant risks. A Dutch study found that colonoscopies carry a small but relevant risk (affecting approximately 3% of patients) of major events requiring hospitalization such as perforation, bleeding, or angina pectoris. They carry a higher rate (approximately 41%) of minor adverse events such as rectal blood loss and a change in bowel habits (10). In addition to these risks, the burden on patients is multifaceted including physical discomfort, psychological distress, and financial costs due to time off-work. Although the process may provide confidence to physicians, it rarely does so for patients. Instead, a diagnosis of exclusion can leave patients confused and reluctant to engage in IBS management (11).

Clearly, there is a need for a new, cost-effective, noninvasive diagnostic test that provides reliable and reproducible positive diagnosis of IBS (7). This could be in combination with blood and stool tests to screen for organic disease for a comprehensive approach. Novel tests on various blood and fecal biomarkers have been trialed (7). However, none alone offer IBS diagnosis with a suitably high positive likelihood ratio and sufficiently low negative likelihood value. So far, a multifaceted approach with the use of symptoms, biomarkers, and psychological markers has been most successful, but it is too complex and time consuming for the primary care setting (7).

Perhaps forgotten in discussions of IBS diagnosis is the work conducted around the millennium by Craine and colleagues (12–14) exploring the use of computerized bowel sound analysis. Craine's team used only short recordings, and relatively simple processing techniques, but had some success in differentiating between IBS and healthy study participants. Their results were less promising with respect to differential diagnosis between IBS and Crohn's disease (13). More recently, Spiegel, Kaneshiro, and colleagues have developed a tool for analysis of bowel sounds for the diagnosis and prognosis of postoperative ileus (15,16). They found a negative predictive value of 81% (16). Their promising results and advances in computing technology prompted us to revisit the use of bowel sound analysis for diagnosing IBS. Our hypothesis was that we could use new signal processing and machine learning–based techniques to develop a positive test for IBS based on bowel sound analysis.

We conducted a preliminary case-control study to both gather data to enable us to characterize IBS through bowel sound features and test the resultant model. The bowel sounds were collected using a belt with an array of vibration sensors. Two periods of recording were made, one when the participants were fasting and one during and after food consumption. We developed and cross-validated a model for characterization of IBS and healthy bowel sounds using the first 31 IBS participants and 37 healthy participants. We went on to test the model on independent data gathered from the next 15 participants in each of the 2 groups. The findings will need to be replicated in a prospective study to confirm their clinical utility; however, this is an important first step.

The intended use of the test is in primary care settings. By offering a positive diagnosis of IBS, we anticipate that it will greatly reduce the number of colonoscopies and replace the process of diagnosis by exclusion. It may be used alongside or instead of Rome IV diagnostic criteria. As with the use of Rome IV, clinicians may also choose to conduct concurrent screening tests for IBD and celiac disease. However, in time, we hope to expand the approach to cover differentiation from organic diseases.

## METHODS

### Study design

We used a diagnostic case-control design for our study. Participants with IBS and those with healthy digestive systems were identified during the recruitment process. Sound recordings for the index test were gathered subsequently as part of the study.

### Participants

#### Recruitment.

We used advertising and media interviews to attract participants, who were recruited consecutively as they responded via phone, e-mail, or online survey and met the eligibility criteria, between May and September 2017. The study was approved by the UWA Human Research Ethics Committee (study RA/4/1/8893—January 25, 2017), and all participants provided informed consent.

#### Eligibility criteria and reference standards.

Eligibility was determined by the use of a short online survey, followed by a more detailed phone survey. Inclusion criteria common to both groups were age 18–65 years, body mass index (BMI) above 18.5, and a good understanding of English.

Inclusion criteria specific to the IBS group were a formal diagnosis of IBS by a general practitioner or gastroenterologist, IBS symptoms for at least 6 months and ongoing IBS symptoms, and the absence of any organic explanation for their IBS symptoms after colonoscopy within the past 10 years (typically in the past 5 years). This was the reference standard for IBS and was confirmed by contacting each patient's doctor. The reference standard did not specify any particular diagnostic symptom criteria, given that some participants may have been diagnosed many years previously, but did include negative colonoscopy results, so as to be highly effective at ruling out organic disease. Anecdotally, we know that a diagnosis of exclusion, such as this, is common in Australia.

Inclusion criteria specific to the healthy group were being asymptomatic at the time of referral and self-reported “generally healthy guts.” This was verified through negative answers to the questions in Supplementary Table 1 (see Supplementary Digital Content 1, http://links.lww.com/CTG/A14).

Exclusion criteria common to both groups were a history of diabetes, eating disorder, kidney disease, neurological disease or damage, current use of opiates or heavy use of nonsteroidal anti-inflammatory drugs, a history of surgery of the GI tract (except for appendectomy or cholecystectomy), a history of organic GI disease including *Helicobacter pylori* infection, stomach or duodenal ulcers (not ulcers due to ulcerative colitis), microscopic colitis, Crohn's disease, ulcerative colitis, cancer anywhere in the GI tract, known intra-abdominal adhesions, and diverticular disease, celiac disease, diagnosed lactose intolerance, or current GI infection.

Once recruited, the participants visited the Marshall Centre at their convenience between June and October 2017 to take part in the study. All were offered a 30 Australian dollars reimbursement to cover travel expenses.

### Index test development and methods

#### Development of the belt.

Abdominal sounds were recorded using a Zoom H6 Handy recorder (Zoom, Tokyo, Japan) attached to 4 piezo-based sensors placed on the 4 quadrants of the abdomen and held in place with stretchy Tubigrip, together referred to as the “belt.” Software was developed for the initial preprocessing that identified bowel sounds for extraction. This system has previously been described following use for recordings of bowel sounds and detection of the migrating motor complex (MMC) (17).

Two clinicians blindly and independently listened to a library of recordings made from the belt. This consisted of 18 putative bowel sounds as identified by our system and 8 sounds categorized as nonbowel sounds (non-GI origin or environmental noise). The clinicians each made a judgment as to whether they were bowel sounds or not based on their clinical experience. There was 100% concordance between both clinicians and the extraction software.

#### Recordings for model development and validation.

Participants fasted from 9 pm the night before recordings and did not consume water from midnight, except to take their regular medications. Recordings took place in a nonclinical setting at the Marshall Centre on the QEII Medical Centre site. Participants sat quietly in armchairs and had access to the internet and reading material. If a participant failed to fast, or complete the full recording session, they were excluded.

Two-hour recordings of bowel sounds began between 9 am and 9.30 am. The participants then had a short break. At the start of the second recording period of 40 minutes, participants received a standard meal: 2 slices of whole meal toast with a portion of butter (or a single banana if unable to tolerate toast) and a glass of water. No adverse events occurred.

#### Signal processing and feature extraction.

**Figure 1:**
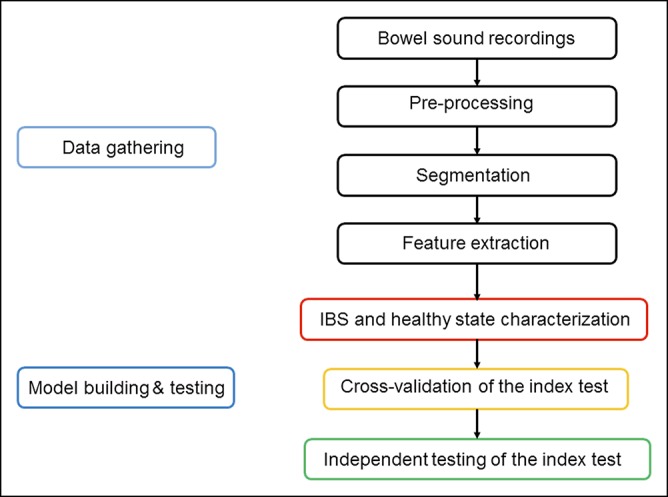
Process flow diagram for gathering data, creation of a machine learning–based classification model, and the 2 stages of testing.

The 160 minutes of recordings, from all 4 channels of each participant, were sampled at 44.1 kHz, equating to approximately 1.6 billion data points. It is impractical to input this amount of data directly into a machine learning model for training. Hence, signal processing was performed to extract features from the data set and reduce the sample's dimensions. The signal processing procedure began with identification of bowel sounds (17). Subsequently, frequency-domain and time-domain features were extracted from each bowel sound, and the approximate location of origin of each bowel sound was determined. The features included many previously identified in the medical (18) and biomedical engineering literature (19) and novel features we developed through our modeling of bowel sound generation (20). The basic process flow of the methodology for gathering the data and creating a machine learning model is presented in Figure 1.

#### Feature analysis, model development, and cross-validation.

Recordings from the first 31 IBS and 37 healthy participants were used to build a model for dichotomous categorization.

The sample sizes selected were limited by time and recruiting constraints. However, similar numbers have been used successfully in the past for proof-of-concept studies (21,22).

Logistical regression analysis was used to identify the optimal array of features most strongly associated with the 2 classes. The model provided an IBS Acoustic Index, with values 0.5 and above predicting IBS, and values below 0.5 predicting healthy, with no indeterminate results. This was compared with the previous clinical diagnosis (reference standard) for each participant to assess accuracy. The model was further fine-tuned using test results from leave-one-out cross-validation (LOOCV). This iterative process was repeated until accuracy plateaued. Internal evaluation of the optimal model's performance was provided by the final LOOCV analysis. The optimal model was subsequently subjected to other k-fold cross-validation techniques and bootstrapping to allow additional evaluation.

### Independent testing

The diagnostic accuracy of the optimal model of the IBS Acoustic Index (the index test) was subsequently tested independently. This independent test was undertaken using the next 15 IBS participants and 15 healthy participants, with performers of the index test blinded to the clinical condition of participants (reference standard). The output of the model (allocation to the IBS or healthy group) for each participant was subsequently compared with the previous clinical diagnosis (reference standard) by another researcher. The reference standard had been undertaken before the study and hence was also performed blinded to the index test results.

Further validation of the cross-validation testing is provided if the independent test results lie inside the confidence interval of the LOOCV results.

### Measures of accuracy

The belt provides a dichotomous output based on the IBS Acoustic Index: a prediction of either IBS or healthy. The results of both the cross-validation and the independent testing were recorded in 2 × 2 contingency tables. We subsequently calculated sensitivity, specificity, negative predictive value, positive predictive value, likelihood ratio for positive test results, and likelihood ratio for negative test results for the index test on each data set.

We investigated whether the accuracy of the test differed for older participants (over 55 years) vs younger participants, different sexes, healthy range vs high BMI, and for the different subtypes of IBS using Fisher exact tests and a significance level of 0.05. Analysis was performed in R (23).

### Impact of food consumption on sounds

We assessed the effect of food consumption on bowel sounds. We investigated changes in the number of bowel sounds per second and the summed amplitude of bowel sounds and looked for significant differences (alpha = 0.05) related to the timing of recording relative to food consumption (fasted or fed) and the presence or absence of IBS and an interaction using a linear mixed model performed using the lme4 package (24) and analyzed with the car package (25) using R (23). For these analyses, individuals were coded as a random variable, and IBS status and food consumption were fixed effects.

## RESULTS

### Participants

**Figure 2:**
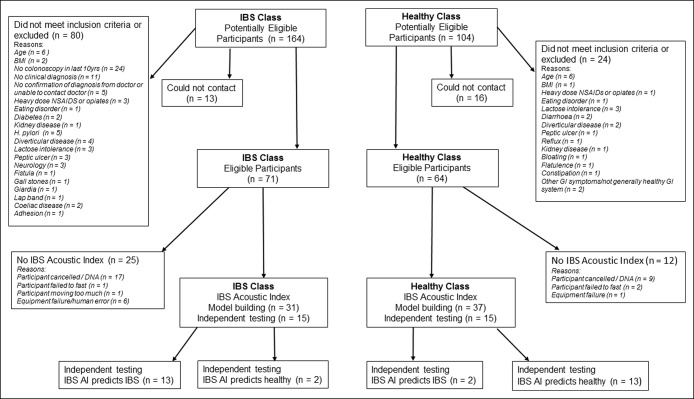
Participant flow for the study.

We received 268 enquiries from potential participants. The longer phone survey and subsequent enquiries to physicians revealed that many did not meet our inclusion criteria. Ultimately, 68 participants undertook the index test for model building, and 30 participants were included in the independent testing. The flow of participants through the study, including reasons for lack of inclusion, exclusion, or lack of index test (IBS Acoustic Index), and the index test results for the 2 groups are provided in Figure 2.

### Demographics

The demographic and clinical characteristics of the healthy and IBS groups are given in Tables 1 and 2, respectively. There were more female participants than males in both groups reflecting their willingness to participate and the fact that women are more likely to report IBS symptoms (1). We had 1 transgender participant, who was in the process of transitioning from female to male. The mean age and mean BMI were similar in the 2 groups. IBS-M was the most common IBS subtype. The subtype was generally based on patient-reported preponderance of symptoms, rather than a clinical diagnosis.

**Table 1:**
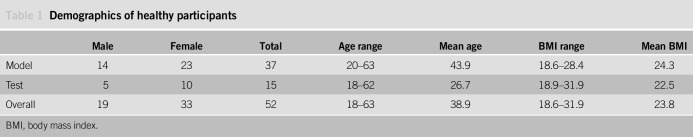
Demographics of healthy participants

**Table 2:**
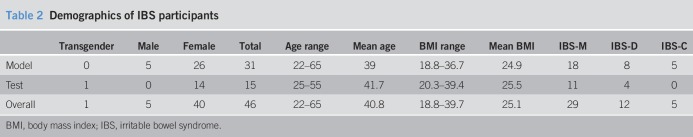
Demographics of IBS participants

### Model and test results

#### Model building and cross-validation.

The optimal model incorporated both time-domain and frequency-domain features and their statistical distributions. The features were derived from both the first recording during the fasted state and the second recording after food consumption and from recordings at all 4 quadrants (26). Two key features were related to the rate of contraction and the motility of the gut, the component interval time, and the burst number. These have been described previously in our mathematical model of bowel sound generation (20). Amplitude during the fasting recording was also an important feature. Amplitude (or more precisely, a sound index describing the summed amplitude) has previously proved useful in determining the cycles of the MMC (17), and it is known that the MMC changes with IBS (27). Other features were derived from the frequency-domain and relate to the spectrum shape and bandwidth, waveform shape, and the subband energy ratios of the bowel sounds.

The LOOCV analysis demonstrated both high sensitivity and specificity for the optimal model (Tables 3 and 4). The overall accuracy, positive likelihood, and negative likelihood of the trained model were 91%, 11.0, and 0.11, respectively (Table 4).

**Table 3:**
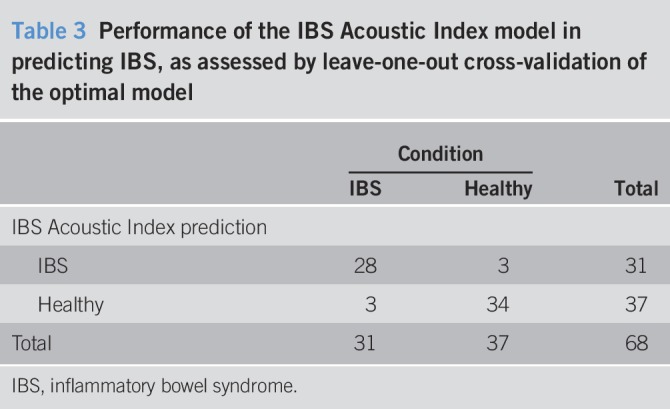
Performance of the IBS Acoustic Index model in predicting IBS, as assessed by leave-one-out cross-validation of the optimal model

**Table 4:**
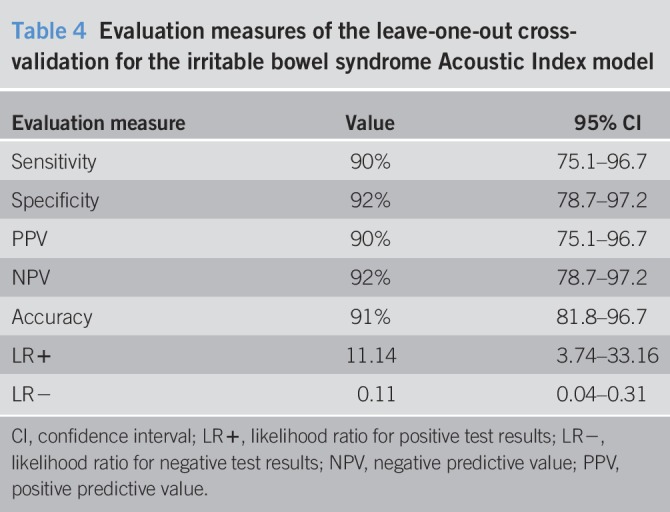
Evaluation measures of the leave-one-out cross-validation for the irritable bowel syndrome Acoustic Index model

Other k-fold cross-validation methods provided overall accuracy values ranging from 0.82 to 0.91 (Tables 5 and 6). The bootstrapping results from 300 repetitions were also similar (Tables 6 and 7).

**Table 5:**
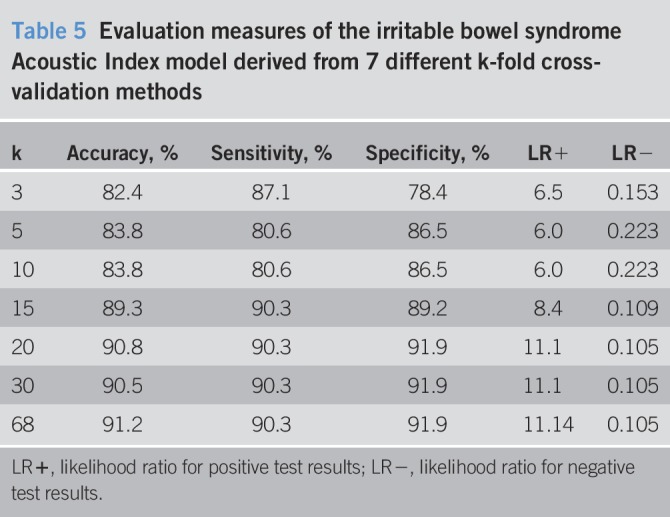
Evaluation measures of the irritable bowel syndrome Acoustic Index model derived from 7 different k-fold cross-validation methods

**Table 6:**
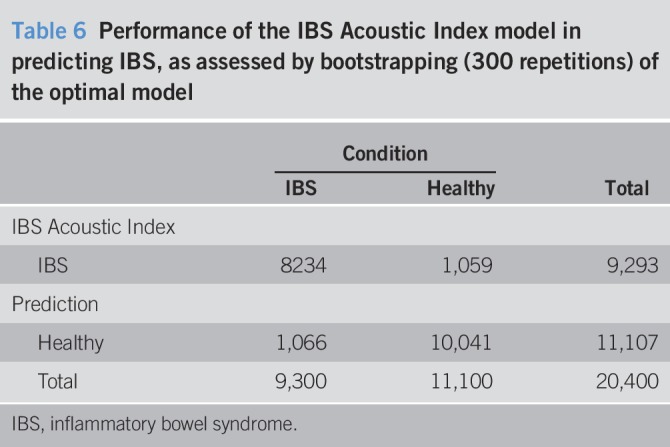
Performance of the IBS Acoustic Index model in predicting IBS, as assessed by bootstrapping (300 repetitions) of the optimal model

**Table 7:**
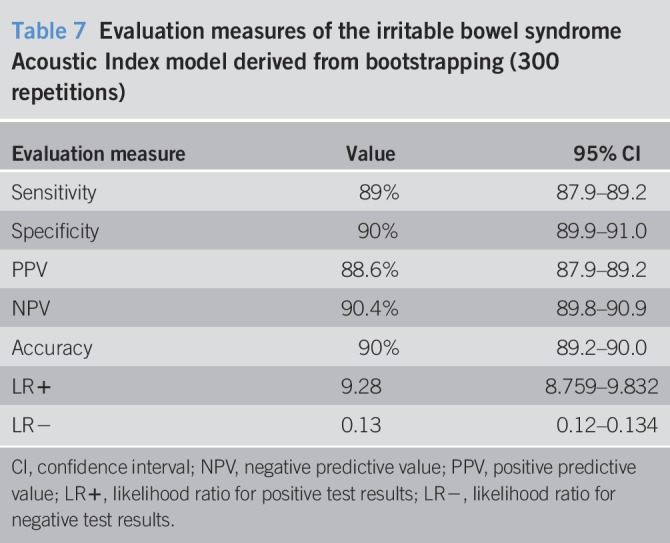
Evaluation measures of the irritable bowel syndrome Acoustic Index model derived from bootstrapping (300 repetitions)

#### Independent test.

**Figure 3:**
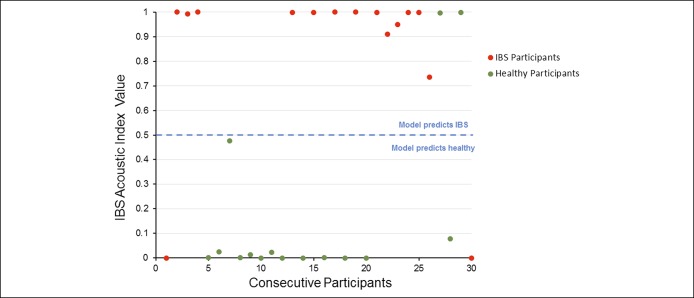
Irritable bowel syndrome Acoustic Index results for the 30 consecutive independent test participants.

The IBS Acoustics Index model performed well on the independent test, demonstrating 87% sensitivity and specificity (Figure 3, Tables 8 and 9). The 2 groups were generally separated by a large margin under the model (Figure 3).

**Table 8:**
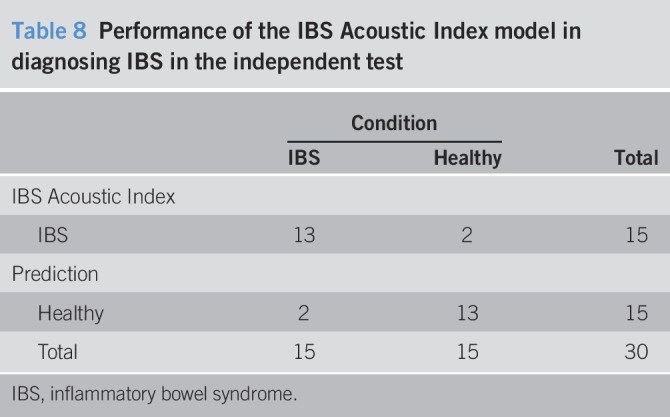
Performance of the IBS Acoustic Index model in diagnosing IBS in the independent test

**Table 9:**
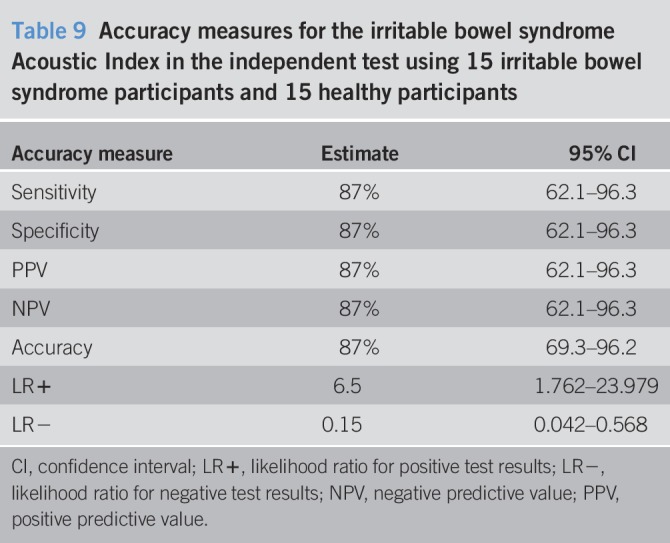
Accuracy measures for the irritable bowel syndrome Acoustic Index in the independent test using 15 irritable bowel syndrome participants and 15 healthy participants

All the accuracy measures were located inside the 95% confidence intervals for the corresponding LOOCV results (Tables 4 and 9).

There was no significant association between sex (male or female), IBS subtype (IBS-D or IBS-M), age (>55 years or not), and BMI (healthy range or 25 and over) and the accuracy rate of the test determined in the independent testing group (Fisher exact test *P* values were 1, 1, 0.25, and 1, respectively). However, it should be noted that the power was extremely low because of small samples of male, IBS-D subtype, and older participants (Tables 1 and 2).

### Impact of food consumption on sounds

**Figure 4:**
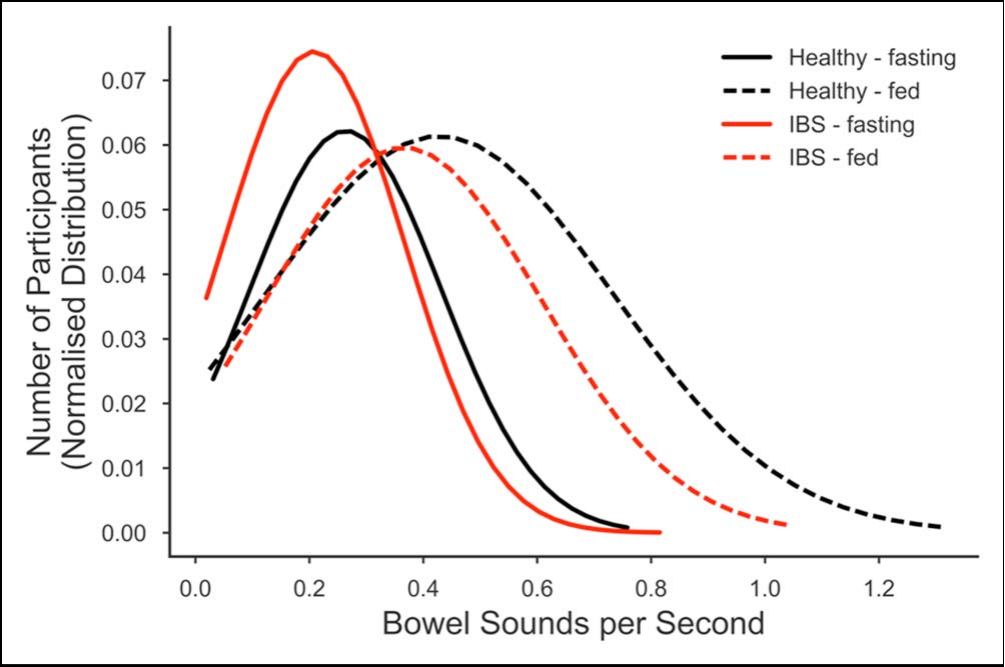
Quantity density distribution. The distribution of 46 irritable bowel syndrome participants and 52 healthy participants across bowel sound quantity density (bowel sounds per second) for the 2 recordings (120 minutes fasting and 40 minutes fed) from the upper right quadrant. The distributions were smoothed to a normal distribution.

Bowel sound density was significantly higher for the healthy individuals than the IBS participants (χ^2^ = 4.04, *P* = 0.045; Figure 4) and after food consumption (χ^2^ = 56.6, *P* < 0.001; Figure 4), but there was no significant interaction between IBS status and the effect of food (χ^2^ = 0.23, *P* = 0.629).

**Figure 5:**
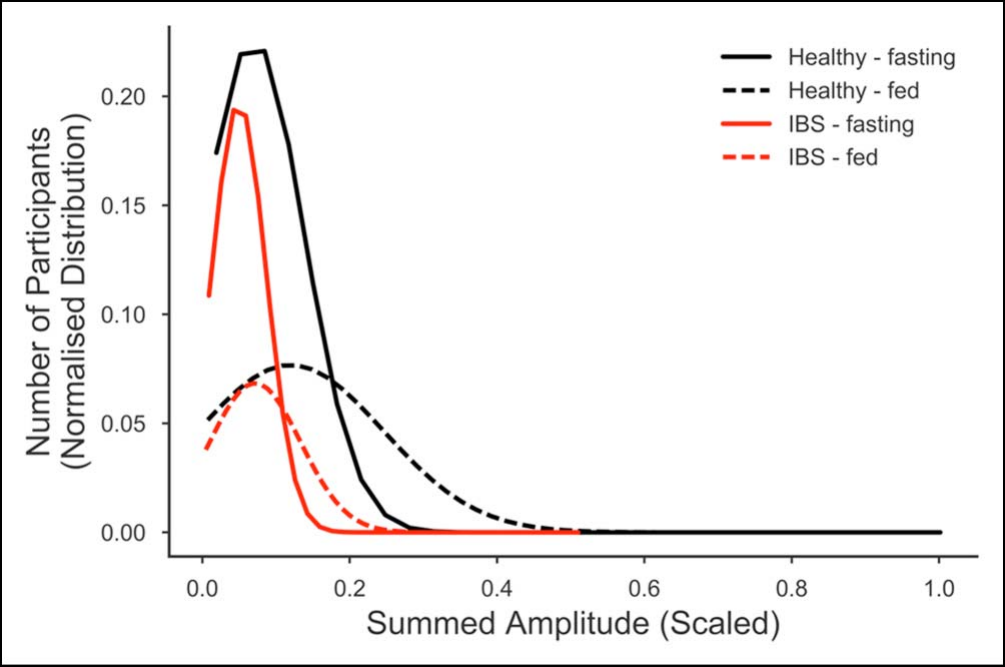
Summed amplitude distribution. The distribition of 46 irritable bowel syndrome and 52 healthy study participants across summed amplitude values for all bowel sounds recorded at the lower right quadrant, during the fed and fasted periods. Summed amplitude values were scaled to compensate for the longer duration of the recording made during the fasting period. The distributions were smoothed to a normal distribution.

Similarly, more higher amplitude sounds were recorded from study participants after eating (Figure 5). There was a significant difference in the summed amplitude between the 2 recording periods (χ^2^ = 22.17, *P* < 0.001) and in IBS individuals relative to the healthy group (χ^2^ = 8.02, *P* = 0.005). Again, we did not find a significant interaction between IBS status and the effect of food (χ^2^ = 3.68, *P* = 0.055).

## DISCUSSION

### A new model

We successfully developed a logistic regression machine learning model that characterized healthy and IBS conditions based on a calculated IBS Acoustic Index derived from 26 bowel sounds features. Both internal validation using the data set used to build the model (LOOCV, other k-fold cross-validation methods and bootstrapping) and external validation (independent testing on new participants) showed high levels of accuracy for the model.

Our approach was innovative with regard to the methods of bowel sound analysis used. Previously, Craine's research group had used short recordings of 2 minutes and only simple sound features. They used the sound-to-sound interval and the proportion of lower frequency sounds for characterization of IBS (12,14). Like Craine et al. (12), we found that there was an increase in bowel sounds after feeding. We also found that there were generally more louder sounds immediately after food consumption. However, our recordings were much longer, and we used a much broader range of features (8 time-domain and 18 frequency-domain features) in the final model (26). Sounds are generated as the contents move through the entire length of the digestive system, especially by the movement of gases through valves (20,28). Given that IBS corresponds with changes in gut motility, the amount of water, and the gases in the contents, it is unsurprising that a variety of sound features characterize this condition. We discovered that 3 key features, the amplitude, burst, and the component interval time, which relate to the MMC and motility, contributed greatly to the model (17,20). Further work is needed to elucidate exactly how all the features relate to the IBS condition.

We also used a method of machine learning, which has been applied widely in other fields, but has not previously been used to build models enabling bowel sound analysis for diagnosis. We used logistic regression–based machine learning (a simple artificial neural network). Other researchers have used other neural networks and wavelets (29), autogressive moving average (AMRA)-based machine learning (30), a hybrid expert system (31), a fuzzy logic system (32), a back propagation neural network (33), and a Bayesian classification method (15) to either identify bowel sounds or for diagnosis of GI conditions.

### Implications for clinical practice

The current gold standard for diagnosis of IBS is a positive diagnosis arrived at using the Rome IV criteria. A positive diagnosis builds confidence in the diagnosis for the patients, improves the clinician-patient relationship, and, hence, sets the groundwork for better management (11). Furthermore, the criteria are less invasive and costly than using colonoscopy to diagnose IBS through exclusion.

However, it has been suggested that although symptom-based diagnostic criteria may be useful for participant recruitment to clinical trials, they are less relevant to clinical practice. The criteria are seen as unwieldy, and validation studies suggest that they only perform modestly well in identification of IBS, especially sensitivity (34).

Researchers have investigated other options, but none are yet considered practical for or have been adopted in widespread clinical practice. In the most recent systematic review of IBS diagnosis, Sood et al. (7) reviewed the accuracy of diagnosing IBS with symptoms, biomarkers, and/or psychological markers. Using any single method provided only modest performance. Approaches combining symptoms and markers, while complex, showed best performance. However, no one combination method met best standards for accuracy of both positive and negative test results.

Our results indicate that sound analysis may offer a new and accurate method for positive diagnosis of IBS. Our belt and model offered both high sensitivity and high specificity for IBS in both internal cross-validation analysis and for independent test cases when differentiating between healthy and IBS individuals. Furthermore, the belt is noninvasive and easy to use.

The intended use of the test is in primary care. It may offer an alternative to the Rome IV diagnostic criteria, but may also be used in combination with them. As with Rome IV, many clinicians would also choose to conduct baseline blood tests (including C-reactive protein) and stool tests (including fecal calprotectin and culture) for exclusion of infections, celiac disease, and inflammatory bowel disease (5). Such an approach provides an alternative to colonoscopy and the process of diagnosis by exclusion for IBS for patients without any red flags. Future work may also allow expansion of the sound analysis approach to diagnosis of organic diseases, such as celiac disease and inflammatory bowel disease.

### Study limitations

Ours was a preliminary study. We used a case-control design including healthy participants. Such studies are generally regarded as less reliable than cross-sectional prospective studies because of spectrum bias. However, it is a good starting point and offers proof of concept that a machine learning approach can provide a new method to characterize GI conditions, such as IBS.

The sample sizes were limited by time and recruiting constraints. However, similar numbers have been used successfully in the past in other proof-of-concept studies to characterize pathological conditions using a machine learning approach (21,22). Our results (especially for external validation) exhibited large confidence intervals indicating statistical uncertainty. However, the fact that we found consistent results with multiple methods of internal validation (multiple k-fold cross-validation analyses and bootstrapping), and independent testing offers increased confidence in the accuracy of our results. These methods included LOOCV, which is a particularly rigorous approach for internal validation.

We included study participants with a range of IBS subtypes and from a wide of ages (18–65 years) and BMI values (18.5–40.5). These are broad, but we cannot currently generalize beyond these ranges. Certainly, bowel motility is known to decrease with age, and this could impact on the utility of the belt for IBS diagnosis in the elderly. In addition, the majority of our participants were younger than 55 years (with only 2 aged 55 years or older in the independent testing group). We had also had limited numbers of male participants (5 in the independent testing group). It would be valuable to reassess the effect of age, sex, and BMI on the accuracy through a larger study with increased power.

Similarly, we had relatively few study participants with self-reported constipation-predominant IBS. We were unable to assess the effect of subtype with a high level of power. In addition, our assessment of subtype was based on self-report of predominant symptoms. Use of the Rome IV criteria for this assessment would be preferable, as would be direct comparison to the Rome IV criteria generally.

We had a wide range of exclusion criteria including comorbidities that may affect bowel motility and GI conditions. Additional research is needed before we can generalize to these groups.

Our belt and model offer hope of a new, more accurate alternative for positive and noninvasive diagnosis of IBS in primary practice. Further cross-sectional prospective studies in the primary care setting with a field prototype are needed for validation, but the sensitivity and specificity demonstrated in this preliminary study are excellent. Expanding the capabilities of the belt to allow differentiation between IBS and other diseases would offer even greater impact.

## CONFLICTS OF INTEREST

**Guarantor of the article:** K. Mary Webberley, BA, MA, PhD.

**Specific author contributions:** X.D.: conducting the study including the signal processing and machine learning components and drafting the manuscript. He has approved the final draft submitted. G.A.: conducting the study, especially hardware development, and editing the manuscript. He has approved the final draft submitted. K.M.W.: planning and conducting the study including coordinating recruitment, interpreting the findings, and drafting the manuscript. She has approved the final draft submitted. A.-J.I.: planning the study, interpreting the findings, and drafting the manuscript. She has approved the final draft submitted. A.O.: planning the study, including providing engineering expertise, and editing the manuscript. He has approved the final draft submitted. B.J.M.: planning the study, interpreting the findings, and editing the manuscript. He has approved the final draft submitted.

**Financial support:** The study was funded by the McCusker Charitable Foundation, but the work was completely independent. The Foundation played no role in the study design, collection, analysis, and interpretation of the data and in the writing of the report.

**Potential competing interests:** B.J.M. received research funding for the project. A.O. received research funding via an agreement between UWA and ECU. X.D., G.A., and K.M.W. were employed by UWA to work on the research project. All the authors are signatories to an intellectual property inventor and contributor agreement with UWA and would benefit from any revenues arising from future successful development and commercialization of the belt.

**Study Highlights**

**WHAT IS KNOWN**

✓ IBS is typically a diagnosis of exclusion after patients undergo colonoscopy to exclude organic disease.
✓ There is a need for a new cost-effective and noninvasive test for positive diagnosis of IBS.

**WHAT IS NEW HERE**

✓ We developed a machine learning–based model to characterize IBS and healthy participants based on bowel sound features.
✓ We achieved proof of concept. Our independent testing demonstrated 87% sensitivity and specificity in identifying IBS.

**TRANSLATIONAL IMPACT**

✓ The results indicate real promise in the use of bowel sound analysis for the positive diagnosis of IBS. The approach could be used in combination with existing screening tests for organic disease.
✓ A positive test result would improve the confidence of patients in the diagnosis, thereby creating a better foundation for management.
✓ The results also bring potential to reduce the burden of unnecessary colonoscopies.
✓ We hope in the future to expand the use of sound analysis to identification of organic disease for even greater clinical impact.

## Supplementary Material

SUPPLEMENTARY MATERIAL
