# Supplementary material for: Noninvasive Diagnosis of Irritable Bowel Syndrome via Bowel Sound Features: Proof of Concept
Source: Clin Transl Gastroenterol. 2019 Mar 22;10(3):e00017. doi: 10.14309/ctg.0000000000000017 (PMC6602778; doi:10.14309/ctg.0000000000000017)
Supplement: SUPPLEMENTARY MATERIAL [file ct9-10-e00017-s001.docx]

## Supplementary Material

**Table 1: Questions to ascertain if healthy participants had good gastrointestinal health**

| **Healthy volunteers provided negative answers to all of the following questions, indicating absence of functional disorders and organic disease.** |
| --- |
| Do you have unusual bouts of nausea or vomiting (not related to an obvious cause, such as food poisoning)? |
| Do you have unusual levels of belching? |
| Do you have unusual levels of bloating? |
| Do you often have constipation with no obvious cause? |
| Do you regularly have diarrhea or loose stools with no obvious cause? |
| Do you regularly have fecal incontinence, pain, or spasms in your anus or problems with defecation? |
| Do you regularly have pain or discomfort somewhere in your GI tract? |
| Do you ever have rectal bleeding? |
| Do you have a feeling of constantly needing to pass stools or do you find yourself frequently straining? |
| Do you find you frequently find yourself urgently needing to have a bowel movement? |
| Do you have diarrhea at night? |
| Do you have mucus or blood in your stools/diarrhea? |
| Have you had significant weight loss, without dieting? |
| Have you ever had any of the following: Helicobacter infection, lots of acid reflux, gastritis, stomach or duodenal ulcers? |
| Do you have and inflamed oesophagus (oesophagitis?) This may be due to either reflux (GORD) or an allergy (oesinophilic oesophagitis). |
